# Prognostic Significance of Chromogranin A Expression in the Initial and Second Biopsies in Metastatic Prostate Cancer

**DOI:** 10.3390/jcm12103362

**Published:** 2023-05-09

**Authors:** Zhuo Huang, Ying Tang, Yuyan Wei, Jingyu Qian, Yifan Kang, Duohao Wang, Miao Xu, Ling Nie, Xueqin Chen, Ni Chen, Qiao Zhou

**Affiliations:** 1 Department of Pathology, West China Hospital, Sichuan University, Chengdu 610041, China; zhuohuang@scu.edu.cn (Z.H.);; 2 Department of Pathology, The First Affiliated Hospital of Chengdu Medical College, Chengdu 610500, China; tangying_chengdu@163.com

**Keywords:** prostate cancer, CgA expression, overall survival, prognosis

## Abstract

Neuroendocrine differentiation (NED) characterized by the expression of neuroendocrine markers, such as chromogranin A (CgA), is frequently observed in advanced prostate cancer (PCa), the prognostic significance of which is still controversial. Here we specifically addressed the issue of the potential prognostic value of CgA expression in advanced-stage PCa patients with distant metastases and its change over time from metastatic hormone-sensitive (mHSPC) to metastatic castration-resistant prostate cancer (mCRPC). CgA expression was assessed immunohistochemically in initial biopsies of mHSPC, as well as in second biopsies of mCRPC in sixty-eight patients, and its correlation with prognosis (together with conventional clinicopathologic parameters) was analyzed using the Kaplan–Meier method and Cox proportional hazard model. We found that CgA expression was an independent adverse prognostic factor for both mHSPC (CgA positivity ≥ 1%, HR = 2.16, 95% CI: 1.04–4.26, *p* = 0.031) and mCRPC (CgA ≥ 10%, HR = 20.19, 95% CI: 3.04–329.9, *p* = 0.008). CgA positivity generally increased from mHSPC to mCRPC and was a negative prognosticator. The assessment of CgA expression may help with the clinical evaluation of advanced-stage patients with distant metastases.

## 1. Introduction

Prostate cancer (PCa) is the second most common malignancy in males and the fifth leading cause of death globally [1]. Although PCa incidence is much lower in Asia, it has also been rising rapidly in East and West Asia [2]. Most prostate cancers are adenocarcinomas, initially being hormone-sensitive prostate cancer (HSPC) with the expression of androgen receptor (AR) and prostate-specific antigen (PSA). Typical first-line androgen deprivation therapy (ADT) usually results in the development of castration-resistant prostate cancer (CRPC) [3]. Although potent AR pathway inhibitors (ARPIs), such as enzalutamide, abiraterone acetate and apalutamide, have therapeutic effects on CRPC, these tumors almost inevitably develop AR-independent pathways to sustain tumor growth after the long-term usage of ARPIs [4,5]. Some CRPCs may develop into treatment-related neuroendocrine prostate cancer (t-NEPC), which is characterized by neuroendocrine carcinoma morphology, expression of neuroendocrine markers, loss of AR expression and independence of AR signaling [6,7]. The most recent WHO classification of prostate cancers considered t-NEPC of the prostate as a unique, independent type [6].

However, it was also observed that a considerable number of prostate adenocarcinomas demonstrated variable neuroendocrine marker expression (also known as neuroendocrine differentiation (NED)) and distant metastasis at the time of diagnosis. These patients are considered to have metastatic hormone-sensitive prostate cancer (mHSPC) with NED, which is responsive to androgen-deprivation therapy and may further develop metastatic castration-resistant prostate cancer (mCRPC). Although it has been generally accepted that NED is related to adverse outcomes [8,9,10], the prognosis of advanced prostate cancer with NED may be different from that of NEPC [7,11].

It is, therefore, worthwhile to further evaluate the prognostic effects of NED in advanced-stage prostate cancers, including mHSPCs and mCRPCs. Among the various neuroendocrine markers employed by different groups, chromogranin A (CgA) is the most specific one compared with synaptophysin (Syn), CD56 and neuron-specific enolase (NSE). In the present study, we specifically evaluated the prognostic significance of CgA expression in the initial biopsies obtained from 68 patients with mHSPCs upon diagnosis and the second prostate biopsies obtained when these patients entered the mCRPC stage.

## 2. Materials and Methods

### 2.1. Patients and Clinicopathological Data

Cases from West China Hospital between 2009 and 2017 were retrospectively collected and reviewed, and sixty-eight patients were selected according to the following inclusion and exclusion criteria. The inclusion criteria required that the patients (1) were diagnosed for the first time with metastatic acinar adenocarcinoma of the prostate via an initial biopsy; (2) received no previous diagnostic or therapeutic procedures for PCa; (3) received maximum androgen blockade treatment, including surgical or medical castration with ADT; (4) were diagnosed with mCRPC according to the guidelines for CRPC diagnosis from the European Association of Urology [12]; and (5) received a second prostate biopsy of mCRPC. Cases were excluded if (1) no remaining tumor existed in the second biopsy, (2) the patient received treatment before the first biopsy or (3) the second biopsy demonstrated t-NEPC features. Each patient served as his own control in this study. All data were collected according to the guidelines of the Ethics Committee of the authors’ institution.

### 2.2. Biopsy and Histopathologic Review

Prostate biopsies were performed using a standard ultrasound-guided transperineal prostate biopsy technique. The initial and second biopsy sections were reviewed independently by two urological pathologists. Histological features, Gleason score (GS) and differences in CgA expression between initial and second biopsy specimens of each patient were assessed.

### 2.3. Immunohistochemistry

Standard immunohistochemical staining for CgA (ZSGB-Bio, Beijing, China, ZA0507, 1:200 dilution) was conducted with negative and positive controls. Unequivocal strong cytoplasmic staining was considered positive staining. The percentage of CgA expression was estimated based on the number of positively stained tumor cells compared with all tumor cells in each needle biopsy specimen, and the average of the percentages of CgA-positive cells in all specimens was the final CgA proportion of the case. Cases with CgA expression > 1% were recorded according to our preliminary analysis and published studies [13,14,15]. Two senior pathologists independently reviewed the slides.

### 2.4. Statistical Analyses

Data on the clinical and pathological variables were summarized using descriptive statistics. The percentages of CgA expression in different GS and ISUP/WHO 2016 grade groups were compared using one-way ANOVA. Overall survival (OS) was defined as the time from HSPC to death (OS^1st^) or that from mCRPC to death (OS^2nd^). OS^1st^ and OS^2nd^ were analyzed by using the Kaplan–Meier method with a log-rank test. A chi-square test was employed to detect the baseline differences between cases with and without CgA expression in the first and second biopsies to estimate the correlation between CgA expression and ISUP/WHO 2016 grading in mHSPC and to examine the difference between CgA expression in mHSPC and mCRPC. The Mann–Whitney U test was applied to assess the impact of CgA expression on CRPC-free survival. The Cox proportional hazard model was employed to investigate the prognostic significance of the clinicopathological variables, as shown by hazard ratios (HRs) with 95% confidence intervals (CIs). All statistical analyses were performed by using SPSS 22.0 (SPSS Inc., Chicago, IL, USA). A *p*-value < 0.05 (two-sided) was considered statistically significant.

## 3. Results

### 3.1. The Clinical Characteristics of the Patient Cohort

The ages of this patient cohort upon diagnosis ranged from 54 to 86, with a median age of 70. The patients were in an advanced stage of prostate cancer and had high Gleason scores and serum PSA levels. The majority of patients (47/68, 69.1%) were diagnosed with a Gleason score of 9 or 10 (WHO grade group 5), and 17 had a Gleason score of 8 (WHO grade group 4). Only four had a Gleason score of 7 (4 + 3; WHO grade group 3). In the second biopsy, 22 cases were not scored and grouped after treatment. The positivity of CgA was not found to correlate with the GS or International Society of Urological Pathology (ISUP)/WHO 2016 grade grouping (*p* = 0.400). In addition, the percentage of CgA expression was also not correlated with the GS or ISUP/WHO 2016 grade groups (*p* ≥ 0.05).

Ninety-seven percent (66/68) of patients presented with bone metastases; the remaining two patients had visceral metastases. Upon first diagnosis, the primary tumors in 25% (17/68) of patients exhibited CgA expression. Clinical and pathological features in the first biopsy and second biopsy are listed in Table 1. By the end point of follow-up, 40 patients (59%) were deceased. The longest follow-up duration was 142.5 months. The median OS^1st^ was 36.9 months (ranging from 3.6 to 142.5 months). The median OS^2nd^ was 23.5 months (ranging from 0.9 months to 63.9 months). There were no significant differences in median age, ISUP/WHO 2016 grade group, castration method, metastasis or serum PSA level between patients with and without CgA expression at the first or second biopsies.

### 3.2. The Tendency of CgA Expression When Developing from mHSPC to mCRPC

**Figure 1:**
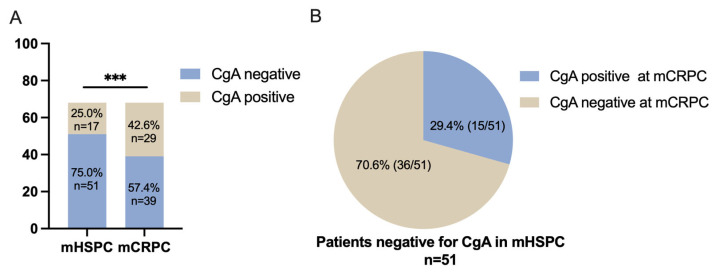
CgA expression status in the present patient cohort. (**A**) Twenty-five percent (17/68) of the cases were positive for CgA in the first biopsy taken at the mHSPC stage. In the second biopsy taken at the mCRPC stage, the percentage increased to 42.6% (*** *p* < 0.001). (**B**) A total of 29.4% (15/51) of cases that were initially negative for CgA at the first biopsy developed a CgA-positive status of mCRPC.

**Figure 2:**
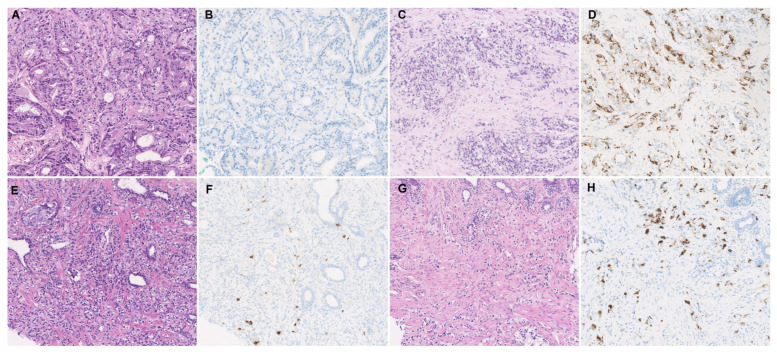
Histological appearance and immunohistochemical staining of CgA. (**A**,**B**) Typical case showing CgA negativity in the initial biopsy of mHSPC, which developed to (**C**,**D**) a CgA-positive status of mCRPC. (**E**,**F**) Typical case with low CgA expression in the initial biopsy of mHSPC that developed to (**G**,**H**) a higher CgA expression status in the second biopsy of mCRPC.

As shown in Table 1 and Figure 1A, CgA expression was found in 25.0% (17/68) of the cases in the initial biopsy at the mHSPC stage. This increased to 42.6% (29/68) in the second biopsy taken at the mCRPC stage (*p* < 0.001). In the initial biopsy, 75% (51/68) of cases were negative for CgA. When these CgA-negative patients (*n* = 51) underwent a second biopsy for mCRPC, 29.4% (15/51) presented with CgA expression (Figure 1B). In those cases with CgA expression in the first biopsy, 30.4% (5/17) of the cases exhibited a prominently increased CgA expression (increased ≥5%) in mCRPC, three cases of which had increased by over 10%. The HE staining and immunostaining of CgA are displayed in Figure 2.

Cases negative for CgA in mHSPC often developed CgA-positive expression together with more Gleason 5 or 4 portions of mCRPC (Figure 2A–D). More than half of the cases with CgA expression exhibited an increased proportion of CgA in mCRPC (Figure 2E–H).

### 3.3. Analysis of the Relationship of Clinicopathological Variables with Survival

Univariate survival analyses using a log-rank test of the clinicopathological variables are summarized in Table 2. The Gleason score and CgA status at the mHSPC and mCRPC stages were significantly associated with both OS^1st^ and OS^2nd^. The overall survival of patients in the ISUP/WHO 2016 grade groups 3, 4 and 5 showed a descending trend; however, this was without statistical significance.

We also examined the effects of CgA expression on CRPC-free survival. CgA expression at the first biopsy correlated with shortened CRPC-free survival (CFS), with a median CFS of 15.93 months in patients negative for CgA vs. 6.17 months in patients positive for CgA (*p* = 0.002, Supplementary Figure S1).

**Figure 3:**
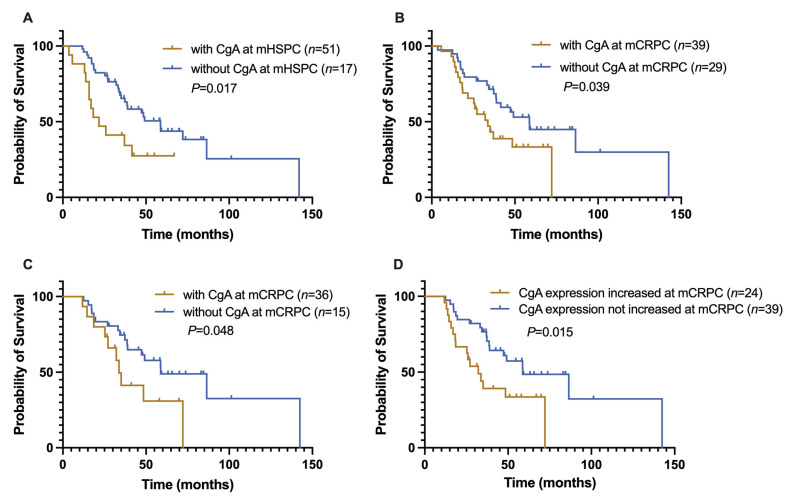
Kaplan–Meier analyses of CgA expression in different groups. (**A**) The OS^1st^ of CgA-positive mHSPC patients was significantly reduced compared with CgA-negative cases (21.7 vs. 58.7 months, *p* = 0.010). (**B**) The OS^1st^ of patients with CgA expression of mCRPC was also markedly decreased (33.7 ± 5.6 months) compared with CgA-negative cases (58.7 months, *p* = 0.039). (**C**) In patients without CgA expression in the first biopsy but with CgA expression of mCRPC (*n* = 51), the OS^1st^ was also shortened (33.7 vs. 58.8, *p* = 0.048). (**D**) In mCRPC patients with increased CgA (increase over 1%) or newly developed CgA expression, the OS^1st^ was prominently reduced compared with those whose CgA expression had not changed (31.1 ± 5.7 vs. 58.8 ± 13.8, *p* = 0.015).

Kaplan–Meier survival analyses are shown in Figure 3. The results indicated that CgA expression at initial diagnosis was associated with a shorter OS^1st^ (21.7 ± 6.1 months vs. 58.7 ± 7.4 months, *p* = 0.017, Figure 3A). CgA expression at the second biopsy was also associated with the median OS^1st^ of this patient cohort (with CgA expression vs. without CgA expression: 33.7 ± 6.1 vs. 58.7 ± 8.8 months, *p* = 0.039, Figure 3B). Cases with newly developed CgA expression of mCRPC (*n* = 15/51) also had a shorter OS^1st^ (33.7 ± 2.4 vs. 58.8 ± 13.9 months, *p* = 0.048, Figure 3C).

### 3.4. Prognostic Significance of the Change in CgA Expression Status of Advanced PCa

We further compared the OS^1st^ of patients (*n* = 24) with increased CgA expression relative to the initial biopsy (*n* = 9) (defined as at least a 1% increase) or with newly developed CgA expression (*n* = 15) of mCRPC to that of patients whose CgA expression status had not changed (*n* = 39). The OS^1st^ of the former was markedly reduced (32.1 ± 5.7 vs. 58.8 ± 13.8 months, *p* = 0.015, Figure 3D).

### 3.5. Hazard Ratio Assessment of Risk Factors of Advanced PCa

Univariate Cox regression analyses also identified the status of CgA of mHSPC and mCRPC and the Gleason score of mHSPC as significant risk factors for OS (Table 3). The multivariate analysis used a Cox proportional risk model that incorporated patient age, Gleason score and CgA expression status (Table 4). The analysis showed that CgA positivity or higher CgA expression of mHSPC or mCRPC was significantly associated with a shortened OS^1st^ or OS^2nd^. The hazard ratio (HR) of CgA expression in the first biopsy for OS^1st^ was 2.16 (95% CI: 1.04–4.26, *p* = 0.031). In the mCRPC stage, the HRs of CgA expression ≥ 10% for OS^1st^ and OS^2nd^ were 20.19 (95% CI: 3.04–329.99, *p* = 0.008) and 5.17 (95% CI: 1.10–33.1, *p* = 0.048), respectively. In addition, the GS was also significantly associated with OS^2nd^ in patients with mHSPC (HR = 2.19, 95% CI: 1.09–4.82, *p* = 0.037) and OS^1st^ in patients with mCRPC (HR = 7.37, 95% CI: 1.84–54.79, *p* = 0.020) (Table 4).

## 4. Discussion

The expression of CgA or other neuroendocrine markers is often observed in prostate adenocarcinomas, particularly after exposure to ADTs. Treatment-related NEPC (t-NEPC) has been adopted as an independent entity characterized by small-cell or large-cell neuroendocrine carcinoma morphology in the most recent WHO classification of prostate cancers [16]. However, the biological features of PCa with the expression of neuroendocrine markers, such as CgA, but not with neuroendocrine carcinoma morphology or t-NEPC features, still needs to be characterized.

The present study of a cohort of 68 typical acinar PCa patients aimed to evaluate the prognostic value of CgA expression and other factors assessed at initial and second biopsies at mHSPC and mCRPC, respectively. Our data indicated that CgA expression (≥1%) was an independent risk factor for shortened OS of those with mHSPC, and the percentage of CgA-positive cells ≥ 10% in biopsy correlated with reduced OS for those with mCRPC. Our results also showed that the percentage of CgA positivity increased as mHSPC progressed to mCRPC, and was also a negative prognosticator.

Among various neuroendocrine markers, CgA showed the highest specificity [17,18]. Studies of CgA expression and its significance in PCa biopsy specimens are limited. Although some investigators did not observe a significant association of CgA expression with OS or disease progression in surgically treated patients with clinically localized PCa [19], other studies showed that the percentage of CgA-positive cells exceeding 1%, 5% or 10% in biopsy specimens of locally advanced PCa was a risk factor for distant metastases, PSA progression-free survival and recurrence [15,20,21]. In a recent study of 35 patients treated with radiotherapy (with or without ADT), an apparently poorer OS and cause-specific survival were observed in cases with focal CgA positivity (>1%), although with marginal statistical significance [13]. The present study specifically addressed the issue of the potential prognostic value of CgA expression in advanced-stage PCa patients with distant metastases and its change over time from mHSPC to mCRPC in a much larger cohort (*n* = 68), and the results suggested the prognostic value of CgA expression for both mHSPC and mCRPC.

Numerous studies also assessed the prognostic value of serum CgA expression in prostate patients, with controversial results [22,23,24,25]. Elevated serum CgA was reported to be an independent prognostic factor for OS and progression-free survival in CRPC patients treated with abiraterone acetate [26] and was related to advanced tumor stage and higher GS [27]. An elevated serum CgA level over three times the upper normal limit in mCRPC patients was a prognostic factor in patients treated with enzalutamide [28]. Although it is more convenient to assess the serum level of CgA, the circulating CgA level can be affected by many factors, such as renal failure, cardiovascular diseases and the use of proton pump inhibitors [29].

Neuroendocrine differentiation in PCa may arise via lineage plasticity, with clonal evolution from either primary PCa or CRPC cells [30]. Single-cell transcriptome sequencing also demonstrated the existence of AR^HIGH^/NE^HIGH^ prostate cancer cells in hormone-naïve prostate cancer cohorts [31]. The precise definition of this clinical state is still lacking, but the expression of NE markers usually confers more aggressive clinical behavior [7,32], which typically bears alterations in *RB1*, *TP53*, *PTEN* and *AR* [33]. PCa with histologic features of adenocarcinoma and the above molecular traits may represent a transition state from typical PCa to PCa with NED [34,35].

In a large single mCRPC dataset, RB1 is the factor that was most strongly associated with poor clinical outcomes [34]. Chromatin binding and transcriptional activity of the RB-repressed E2F1 are highly dependent on lysine-specific demethylase 1A (LSD1) in *RB1*-deficient CRPC, and RB1 inactivation enables CRPC tumors to be sensitive to LSD1 inhibitor [36].

Dual loss of *TP53* and *RB1* was reported to promote lineage plasticity and was correlated with worse clinical outcomes in patients with metastatic PCa [34]. In RB1/TP53-silenced LNCAP*^PTEN−/−^* cells, epigenetic reprogramming factors, such as EZH2 and SOX2, were repressed, which may provide stem-like status for lineage plasticity [37]. LNCAP*^TP53−/−; RB1−/−^* cells exhibited exuberant proliferation, loss of G1/S checkpoint, replication stress, repressed AR signaling and stem-like features without NE activity [38]. *RB1* and *TP53* dual-knockout (DKO) cell lines also showed a reduced enzalutamide response duration.

*PTEN* plays a key role in prostate tumorigenesis. *PTEN* loss was correlated with higher GSs, poorer prognosis and increased metastasis potentials [39]. *PTEN* deletion induces prostatic intraepithelial neoplasia in mice.

Genome-wide DNA methylation analysis of CRPC and CRPC-NED also revealed significant epigenetic dysregulations that may contribute to NED [30]. Promoter methylation of SAM pointed domain containing ETS transcription factor (SPDEF), which encodes prostate-derived ETS factor (PDEF) and functions as a transcription activator and cell differentiation regulator, was observed in neuroendocrine cancer cell line NCI-H660 [40].

In RB1/TP53-silenced LNCAP^PTEN^*^−/−^* cells, epigenetic reprogramming factors, such as enhancer of zeste 2 polycomb repressive complex 2 subunit (EZH2) and SRY-Box transcription factor 2 (SOX2), are repressed. The suppression of these factors might help provide a stem-like environment for lineage plasticity in the context of genomic alterations involving the loss of RB1, TP53 and PTEN functions [37]. The histone methyltransferase EZH2 is prominently overexpressed in CRPC-NED [30], and the EZH2-repressed target genes are simultaneously downregulated, including WNT signaling and homeobox factor-encoding genes. The roles of EZH2 in prostate cancer with neuroendocrine features were investigated in several studies. In mouse model CRPC with neuroendocrine differentiation, overexpressed N-Myc interacts with the SET domain of EZH2 and SUZ12 subunit of PRC2. EZH2 together with N-Myc interacts with AR to form the N-Myc/AR/EZH2-PRC2 complex, inducing NED and the abrogation of AR signaling [41].

In enzalutamide-induced neuroendocrine differentiation, EZH2 interacts with lncRNA-p21 instead of HOTAIR due to competitive binding. LncRNA-p21 promotes the interaction of EZH2 and Serine/Threonine Kinase 1 (AKT), which phosphorylates EZH2 S21 and activates signal transducer and activator of transcription 3 (STAT3) [42,43]. In addition, EZH2 reverses the inactivation of Forkhead Box O1 (FOXO1) and promotes NED via repressing miR-708 [44]. EZH2 epigenetically represses Thrombospondin 1 (TSP1) to relieve the inhibition of angiogenesis [45]. In vitro and animal studies have indicated that EZH2 inhibitors, such as GSK343, could be a promising therapy for prostate cancer with neuroendocrine differentiation [30,37,41].

## 5. Conclusions

In summary, our data showed that CgA expression at either the mHSPC or mCRPC stages was correlated with prognosis, and its assessment may help with the clinical evaluation of patients with metastatic PCa.

## Figures and Tables

**Table 1:** Clinicopathological features of the patient cohort.

Characteristics	Initial Biopsy				Repeated Biopsy			
	Total	With CgA Expression	Without CgA Expression		Total	With CgA Expression	Without CgA Expression	
	(*n* = 68)	(*n* = 17)	(*n* = 51)	*p*	(*n* = 68)	(*n* = 29)	(*n* = 39)	*p*
**Median age (** **range)**	68 (51–84)	67 (51–83)	69 (52–84)	0.228	70 (54–86)	67 (54–85)	70 (54–86)	0.136
**ISUP/WHO** **2016 grade group**	*n* (%)	*n* (%)	*n* (%)		*n* (%)	*n* (%)	*n* (%)	
Group 3	4 (5.9)	2 (11.8)	2 (3.9)	0.400	2 (2.9)	1 (3.4)	1 (2.6)	0.977
Group 4	17 (25)	3 (17.6)	14 (27.5)		3 (4.4)	1 (3.4)	2 (5.1)	
Group 5	47 (69.1)	12 (70.6)	35 (68.6)		41 (60.3)	18 (62.1)	23 (59.0)	
Not grouped					22 (32.4)	9 (31.1)	13 (33.3)	
**Gleason score**								
<9	21 (30.9)	5 (29.4)	16 (31.4)	0.880	5 (7.4)	2 (6.9)	3 (7.7)	0.967
≥9	47 (69.1)	12 (70.6)	35 (68.6)		41 (60.3)	18 (62.1)	23 (59.0)	
Not grouped					22 (32.4)	9 (31)	13 (33.3)	
**Castration**								
Surgery	22 (32.4)	8 (47.1)	14 (27.5)	0.134	22 (32.4)	10 (34.5)	12 (30.8)	0.746
Drugs	46 (67.6)	9 (52.9)	37 (72.5)		46 (67.6)	19 (65.5)	27 (69.2)	
**Metastasis**								
Visceral metastasis	2 (2.9)	1 (5.9)	1 (2.0)	0.440	2 (2.9)	1 (3.4)	1 (2.6)	1.000
Bone metastasis	66 (97.1)	16 (94.1)	50 (98.0)		66 (97.1)	28 (96.6)	38 (97.4)	
**PSA (ng/mL)**								
≥100	43 (63.2)	10 (58.8)	33 (64.7)	0.663	43 (63.2)	15 (51.7)	28 (71.8)	0.090
<100	25 (36.8)	7 (41.2)	18 (35.3)		25 (36.8)	14 (48.3)	11 (28.2)	

Abbreviations: ISUP/WHO, International Society of Urological Pathology/the World Health Organization; CgA, chromogranin A; PSA, prostate-specific antigen; *n*: number of cases; *p*-values refer to differences in the clinicopathological parameters of enrolled patients.

**Table 2:** Univariate survival analysis of OS^1st^ and OS^2nd^.

Variates	OS^1st^ (Months)		OS^2nd^ (Months)	
	Median (95% CI)	*p*	Median (95% CI)	*p*
**Residual tumor of mCRPC (%)**				
≥40% vs. <40%	47.8 (25.5–70.0) vs. 58.7 (43.5–73.8)	0.487	23.6 (11.4–35.8) vs. 36.5 (25.3–47.7)	0.100
**Age at first diagnosis (y)**				
≥68 vs. <68	49.1 (35.6–62.6) vs. 41.5 (15.1–67.9)	0.415	25.9 (11.3–40.4) vs. 29.5 (13.3–45.6)	0.822
**Age at mCRPC status (y)**				
≥70 vs. <70	50.0 (35.5–64.5) vs. 41.5 (22.4–60.6)	0.129	25.9 (10.8–41.0) vs. 29.5 (19.3–39.6)	0.824
**GS at mHSPC status**				
≥9 vs. <9	41.3 (26.1–56.5) vs. 86.5 (35.1–137.8)	**0.015**	22.2 (12.6–31.8) vs. 44.0	**0.004**
**GS at mCRPC status**				
≥9 vs. <9	49.1 (25.3–73.0) vs. 58.7 (0–120.9)	0.625	32.3 (25.9–38.7) vs. 46.4 (27.4–65.3)	0.415
**ISUP/WHO 2016 grade group**				
Group 3 vs. group 4 vs. group 5	67.3 (38.2–96.4) vs. 64.7 (49.1–80.4) vs. 51.8 (32.9–70.8)	0.128	50.9 (28.8–72.9) vs. 42.2 (32.2–52.1) vs. 25.8 (20.2–31.3)	0.074
**CgA status of mHSPC**				
Positive vs. negative	21.7 (9.7–33.7) vs. 58.7 (44.2–73.1)	**0.017**	12.1 (7.4–16.8) vs. 33.3 (23.4–43.3)	0.103
CgA ≥ 10% vs. CgA < 10%	15.8 (4.9–26.8) vs. 48.4 (25.6–71.3)	**<0.001**	10.9 (0.7–21.0) vs. 31.9 (21.7–42.1)	**0.001**
**CgA status of mCRPC**				
Positive vs. negative	35.0 (11.5–58.5) vs. 58.7 (39.6–77.8)	**0.025**	14.7 (8.5–20.8) vs. 37.3 (23.4–51.2)	**0.023**
CgA ≥ 10% vs. CgA < 10%	20.5 (14.0–27.0) vs. 51.1 (39.8–62.4)	**0.007**	12.1 (9.7–14.5) vs. 31.9 (21.2–42.6)	**0.001**
**Castration**				
Surgical vs. medical	47.8 (30.0–66.0) vs. 49.1 (35.4–62.8)	0.743	25.2 (7.5–43.0) vs. 27.0 (16.6–37.3)	0.459
**Metastasis at mCRPC**				
Visceral metastasis vs. bone metastasis only	59.9 (14.9–104.9) vs. 48.4 (30.0–66.9)	0.843	49.2 (22.2–76.2) vs. 25.2 (12.9–37.6)	0.600
**Serum PSA at mCRPC (ng/mL)**				
≥100 vs. <100	41.5 (25.4–57.6) vs. 58.8 (35.0–82.6)	0.332	31.3 (7.9–54.8) vs. 25.2 (15.8–34.7)	0.697

Abbreviations: OS^1st^, the OS from first diagnosis to death; OS^2nd^, the OS from the second biopsy or the diagnosis of mCRPC to death; CI, confidence interval; mCRPC, metastatic castration-resistant prostate cancer; mHSPC, hormone-sensitive prostate cancer; GS, Gleason score; CgA, chromogranin A; PSA, prostate-specific antigen. The *p*-values < 0.05 are in bold.

**Table 3:** Univariate Cox regression analyses of OS^1st^ and OS^2nd^.

Variables	Data Type	OS^1st^ (Months)		OS^2nd^ (Months)	
		HR (95% CI)	*p*	HR (95% CI)	*p*
Age at mHSPC status	Continuous	0.99 (0.96–1.03)	0.588	1.00 (0.97–1.04)	0.754
Age at mCRPC status	Continuous	0.97 (0.94–1.00)	0.058	1.00 (0.96–1.03)	0.833
Tumor% at mCRPC status	Continuous	1.00 (0.99–1.01)	0.773	1.00 (1.00–1.01)	0.381
Gleason score at mHSPC status	≥9 vs. <9	2.048 (1.02–4.48)	0.055	2.26 (1.13–4.97)	**0.015**
CgA status of mHSPC	≥1% vs. <1%	2.28 (1.14–4.58)	**0.020**	1.75 (0.89–3.48)	0.108
Proportion of CgA at mHSPC status	≥10% vs. <10%	4.29 (1.89–9.73)	**<0.001**	2.81 (1.26–6.26)	**0.012**
CgA status of mCRPC	≥1% vs. <1%	1.77 (1.07–2.95)	**0.028**	1.78 (1.08–2.95)	**0.025**
Proportion of CgA at mCRPC status	≥10% vs. <10%	2.27 (1.24–4.16)	**0.008**	2.84 (1.53–5.29)	**0.001**
Castration method	Surgery vs. medicine	1.09 (0.65–1.82)	0.744	1.21 (0.73–2.01)	0.460
Metastasis of mCRPC	Viscera vs. bone	1.11 (0.40–3.08)	0.843	0.76 (0.27–2.12)	0.601
Serum PSA level of mCRPC (ng/mL)	≥100 vs. <100	1.32 (0.75–2.31)	0.334	1.15 (0.64–1.93)	0.698

Abbreviations: OS^1st^, the OS from first diagnosis to death; OS^2nd^, the OS from the second biopsy or the diagnosis of mCRPC to death; mHSPC, metastatic hormone-sensitive prostate cancer; mCRPC, metastatic castration-resistant prostate cancer; CgA, chromogranin A; PSA, prostate-specific antigen. The *p*-values < 0.05 are in bold.

**Table 4:** Multivariate Cox proportional hazards regression analyses of OS^1st^ and OS^2nd^.

Variates	Data Type	OS^1st^ (Months)		OS^2nd^ (Months)	
		HR (95% CI)	*p*	HR (95% CI)	*p*
**For mHSPC**					
Age at mHSPC status	Continuous	1.00 (0.96–1.04)	0.804	1.00 (0.96–1.04)	0.994
GS at first biopsy	≥9 vs. <9	1.97 (0.98–4.33)	0.070	2.19 (1.09–4.82)	**0.037**
CgA expression	≥1% vs. <1%	2.16 (1.04–4.26)	**0.031**	1.67 (0.81–3.23)	0.145
**For mCRPC**					
Age at mCRPC status	Continuous	1.05 (0.97–1.17)	0.265	1.03 (0.95–1.12)	0.533
GS at second biopsy	≥9 vs. <9	7.37 (1.84–54.79)	**0.020**	4.92 (1.33–31.52)	0.054
CgA expression	≥10% vs. <10%	20.19 (3.04–329.9)	**0.008**	5.17 (1.10–33.13)	**0.048**

Abbreviations: OS^1st^, the OS from first diagnosis to death; OS^2nd^, the OS from the second biopsy or the diagnosis of mCRPC to death; HR, hazard ratio; mHSPC, metastatic hormone-sensitive prostate cancer; mCRPC, metastatic castration-resistant prostate cancer; CgA, chromogranin A; GS, Gleason score. The *p*-values < 0.05 are in bold.

## Data Availability

Data of the study are available from the corresponding authors upon reasonable request.

## Supplementary Materials

The following supporting information can be downloaded from https://www.mdpi.com/article/10.3390/jcm12103362/s1, Figure S1: The CRPC-free survival in CgA-positive and CgA-negative patients in the first biopsy.
