# Supplementary material for: Prognostic Significance of Chromogranin A Expression in the Initial and Second Biopsies in Metastatic Prostate Cancer
Source: J Clin Med. 2023 May 9;12(10):3362. doi: 10.3390/jcm12103362 (PMC10219341; doi:10.3390/jcm12103362)
Supplement: Supplementary file 1 [file jcm-12-03362-s001.zip › jcm-2220198-supplementary.pdf]

Supplementary material

Prognostic Significance of Chromogranin A Expression in the Initial and Second Biopsies in Metastatic Prostate Cancer

Zhuo Huang [1,†], Ying Tang [2,†], Yuyan Wei [1], Jingyu Qian [1], Yifan Kang [1], Duohao Wang [1], Miao Xu [1], Ling Nie [1], Xueqin Chen [1], Ni Chen [1,*] and Qiao Zhou [1,*]

[1] Department of Pathology, West China Hospital, Sichuan University, Chengdu 610041, China
[2] Department of Pathology, The First Affiliated Hospital of Chengdu Medical College, Chengdu 610500, China
* Correspondence: chenni1@163.com (N.C.); zhou_qiao@hotmail.com (Q.Z.)
† These authors have contributed equally to this work and share first authorship.

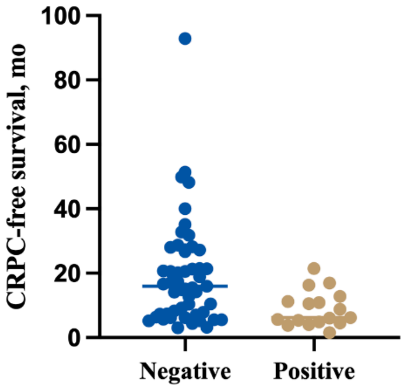

Supplementary Figure S1. The CRPC-free survival in CgA-positive and CgA-negative patients in the first biopsy. The CRPC-free survival (from mHSPC to mCRPC) was significantly reduced in CgA positive patients at mHSPC (p = 0.002).
